# Emodin Ameliorates LPS-Induced Acute Lung Injury, Involving the Inactivation of NF-κB in Mice

**DOI:** 10.3390/ijms151119355

**Published:** 2014-10-24

**Authors:** Min Xiao, Tao Zhu, Wei Zhang, Tao Wang, Yong-Chun Shen, Qiong-Fang Wan, Fu-Qiang Wen

**Affiliations:** 1 Division of Pulmonary Diseases, State Key Laboratory of Biotherapy of China, Department of Respiratory Medicine, West China Hospital of Sichuan University, Chengdu 610041, China; E-Mails: xiaomin_wcms@163.com (M.X.); zhutao15452@163.com (T.Z.); wangtaowcms@163.com (T.W.); shenyongchun2014@126.com (Y.-C.S.); wanqiongfang2012@163.com (Q.-F.W.); 2 Respiratory Medicine, the First Affiliated Hospital of Chengdu Medical College, Chengdu 610500, China; E-Mail: zhangweicdmc@126.com

**Keywords:** emodin, acute lung injury (ALI), acute respiratory distress syndrome (ARDS), lipopolysaccharide (LPS), E-selectin, MCP-1, nuclear factor-κB (NF-κB)

## Abstract

Acute lung injury (ALI) and its severe manifestation of acute respiratory distress syndrome (ARDS) are well-known illnesses. Uncontrolled and self-amplified pulmonary inflammation lies at the center of the pathology of this disease. Emodin, the bio-active coxund of herb *Radix rhizoma* Rhei, shows potent anti-inflammatory properties through inactivation of nuclear factor-κB (NF-κB). The aim of this study was to evaluate the effect of emodin on lipopolysaccharide (LPS)-induced ALI in mice, and its potential bio-mechanism. In our study, BALB/c mice were stimulated with LPS to induce ALI. After 72 h of LPS stimulation, pulmonary pathological changes, lung injury scores, pulmonary edema, myeloperoxidase (MPO) activity, total cells, neutrophils, macrophages, TNF-α, IL-6 and IL-1β in bronchoalveolar lavage fluid (BALF), and MCP-1 and E-selectin expression were notably attenuated by emodin in mice. Meanwhile, our data also revealed that emodin significantly inhibited the LPS-enhanced the phosphorylation of NF-κB p65 and NF-κB p65 DNA binding activity in lung. Our data indicates that emodin potently inhibits LPS-induced pulmonary inflammation, pulmonary edema and MCP-1 and E-selectin expression, and that these effects were very likely mediated by inactivation of NF-κB in mice. These results suggest a therapeutic potential of emodin as an anti-inflammatory agent for ALI/ARDS treatment.

## 1. Introduction

Acute lung injury (ALI) and its severe manifestation, acute respiratory distress syndrome (ARDS), are well-known fatal diseases with an extremely high morbidity rate (35% to 50%) in critically ill patients [1,2,3]. According to reports, approximately 190,000 new cases were diagnosed in the United States per year [1,2,4,5,6,7,8,9]. The multiple etiologies, including severe sepsis, pneumonia, lung abscess and severe burn, cause uncontrolled and self-amplified pulmonary inflammation which lie at the center of the pathology of ALI/ARDS [2,10,11,12,13,14,15,16]. Studies confirmed that alveolar epithelium and pulmonary endothelium are the primary injury targets of this disease [2,10,11,12,13,14,15,16]. Although great progress has been made in understanding the pathogenesis and pathophysiology of ALI/ARDS, the treatments have still been limited.

Nuclear factor-κB (NF-κB), a widely expressed nuclear transcription factor, contributes substantially to the regulation of multiple important physiopathological processes, such as inflammation, apoptosis, oxidative stress and tumor proliferation and metastasis [15,17,18,19,20]. Furthermore, the NF-κB signaling pathway plays a critical role in the inflammatory processes in ALI/ARDS [15,17,18,19,20]. Reports demonstrated that the severity of inflammation in ALI/ARDS was greatly reduced by inhibition of the NF-κB signaling pathway [15,17,18,19,20]. Further, multiple studies demonstrated that the NF-κB signaling pathway is a promising therapeutic target of ALI/ARDS and other inflammatory diseases, including arthritis, pulmonary fibrosis, asthma and hepatitis [17,18,21].

*Radix rhizoma* Rhei, a popular herb in traditional Chinese medicine and other folk medicine in East Asian countries for more than a thousand years, was widely used for the treatment of diarrhoea, dysentery and fever. Emodin was the main active component of *Radix rhizoma* Rhei. Recent studies confirmed the anti-inflammatory value of emodin in several conditions, such as pancreatitis, atherosclerosis, asthma, hepatitis and sepsis [22,23,24,25,26,27,28]. Furthermore, it has been found that the anti-inflammatory effect of emodin results from inhibition of the NF-κB signaling pathway [22,23,24,25,26,27,28]. However, the value of emdoin in LPS-induced pulmonary inflammation is still unclear. Thus, the purpose of this study was to elucidate whether emdoin could ameliorate LPS-induced ALI/ARDS through inhibition of the NF-κB signaling pathway in mice.

## 2. Results

### 2.1. Emodin Ameliorates Pulmonary Inflammation and Pulmonary Edema in Lipopolysaccharide (LPS)-Induced Acute Lung Injury (ALI)

**Figure 1:**
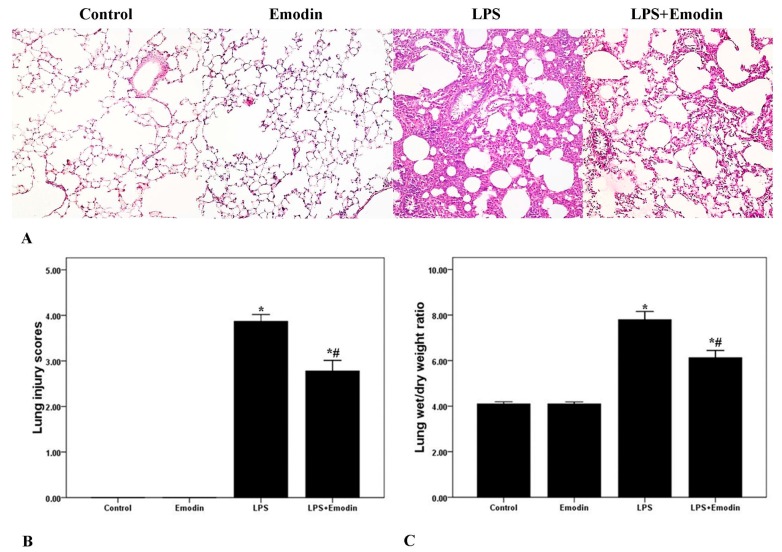
Emodin ameliorates pulmonary inflammation and pulmonary edema in lipopolysaccharide (LPS)-induced acute lung injury (ALI). (**A**) After 72 h interventions, mice were sacrificed and their right lower lungs were fixed. Then, tissue sections were stained with hematoxylin and eosin (H&E). The figure demonstrates a representative view (×200) from each group; (**B**) Severity of lung injury was analyzed by the lung injury scoring system; (**C**) The left lower lungs were obtained to assess wet to dry (W/D) ratio of lung. Each bar represents the mean ± SD of 10 mice. * *p* < 0.05 compared with Control. # *p* < 0.05 compared with LPS.

To observe the pathological changes of the lung tissues, hematoxylin and eosin (H&E) staining and a lung injury score system were utilized in our study. As shown in Figure 1A, the lung tissues from the LPS group demonstrated significantly pathological alterations, including notable inflammatory cells infiltration, interstitial and intra-alveolar edema and patchy hemorrhage, inter-alveolar septal thickening, hyaline membrane formation and some collapsed alveoli. Otherwise, non-cardiogenic pulmonary edema was another critical feature of ALI/ARDS. To evaluate the severity of pulmonary edema, wet to dry ratio (W/D) was obtained in our study. As shown in Figure 1C, W/D was markedly increased after LPS injection. Meanwhile, the lung injury scores and W/D were both markedly attenuated by emodin intervention. Additionally, no difference in lung injury scores and W/D was found between the Control group and the Emodin group.

### 2.2. Emodin Decreases Cell Counts and Inflammatory Mediators in Bronchoalveolar Lavage Fluid (BALF) in LPS-Induced ALI

**Figure 2:**
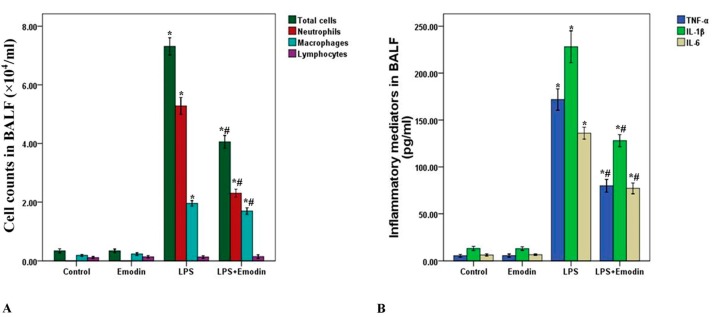
Emodin decreases cell counts and inflammatory mediators in bronchoalveolar lavage fluid (BALF) in LPS-induced ALI. Three days after interventions, mice were sacrificed and their lungs were lavaged. (**A**) Cells in BALF were collected and cytospin preparations were made. Total cells, neutrophils, macrophages and lymphocytes in BALF were measured; (**B**) TNF-α, IL-6 and IL-1β in BALF were analyzed by ELISA. Each bar represents the mean ± SD of 10 mice. * *p* < 0.05 compared with Control. # *p* < 0.05 compared with LPS.

To further evaluate the anti-inflammatory property of emodin, cell counts and inflammatory mediators, including TNF-α, IL-6 and IL-1β in bronchoalveolar lavage fluid (BALF) were measured in our study. As shown in Figure 2, the number of total cells, neutrophils, macrophages and the level of TNF-α, IL-6 and IL-1β in BALF were significantly increased 3 days after LPS stimulation. However, emodin largely reduced the number of total cells, neutrophils, macrophages and the level of TNF-α, IL-6 and IL-1β in BALF. In addition, no difference in cell counts and inflammatory mediators in BALF was found between the Control group and Emodin group. Further, differences in lymphocyte levels in BALF was not found among all groups (Figure 2A).

### 2.3. Emodin Inhibits Myeloperoxidase (MPO) Activity in LPS-Induced ALI

**Figure 3:**
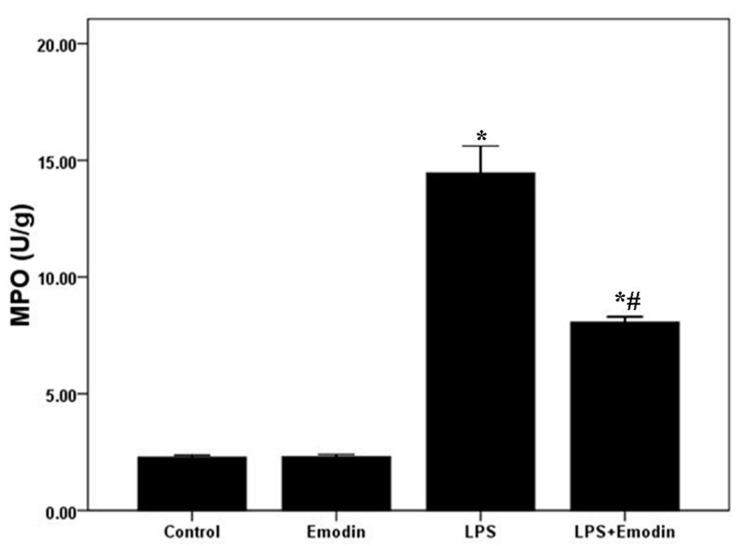
Emodin inhibits myeloperoxidase (MPO) activity in LPS-induced ALI. After 72 h interventions, mice were sacrificed, and their lungs were removed. MPO activity was measured to assess the accumulation and activation of neutrophils in the lung tissues. Each bar represents the mean ± SD of 10 mice. * *p* < 0.05 compared with Control. # *p* < 0.05 compared with LPS.

Myeloperoxidase (MPO) activity was used to assess the activation and accumulation of neutrophils in the lung tissues in the current study. After 72 h of LPS injection, MPO activity was significantly enhanced. Meanwhile, MPO activity was substantially inhibited by emodin (Figure 3). No difference in MPO was observed between Control group and Emodin group.

### 2.4. Emodin Reduces E-Selectin and MCP-1 Expression in Lung Tissues in LPS-Induced ALI

**Figure 4:**
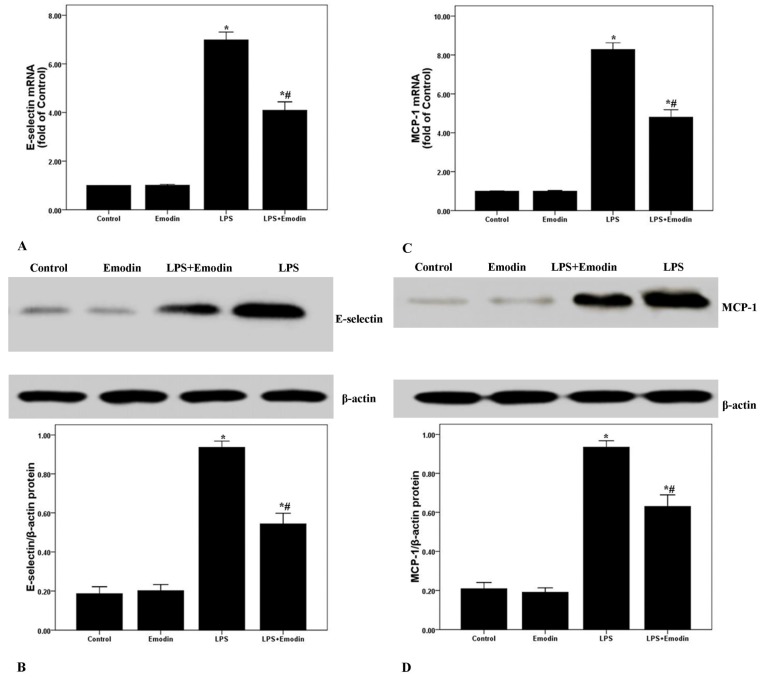
Emodin reduces E-selectin and MCP-1 expression in the lung tissues in LPS-induced ALI. After 72 h interventions, mice were exsanguinated and their lungs were removed. (**A**,**C**) qPCR was performed to analyze E-selectin and MCP-1 mRNA expression in the lung tissues; (**B**,**D**) Western blotting was performed to measure E-selectin and MCP-1 protein expression in the lung tissues. Each bar represents the mean ± SD of 10 mice. * *p* < 0.05 compared with Control. # *p* < 0.05 compared with LPS.

E-Selectin and MCP-1 played the important roles in the pathogenesis of ALI/ARDS. After 72 h of LPS injection, the mRNA and protein expression of E-selectin and MCP-1 were noticeably increased. Nevertheless, the mRNA and protein expression of E-selectin and MCP-1 were remarkably inhibited by emodin (Figure 4). Additionally, no difference in the mRNA and protein expression of E-selectin and MCP-1 was found between the Control group and the Emodin group.

### 2.5. Emodin Inhibits LPS-Induced NF-κB Activation and DNA Binding Activity in Lung in LPS-Induced ALI

**Figure 5:**
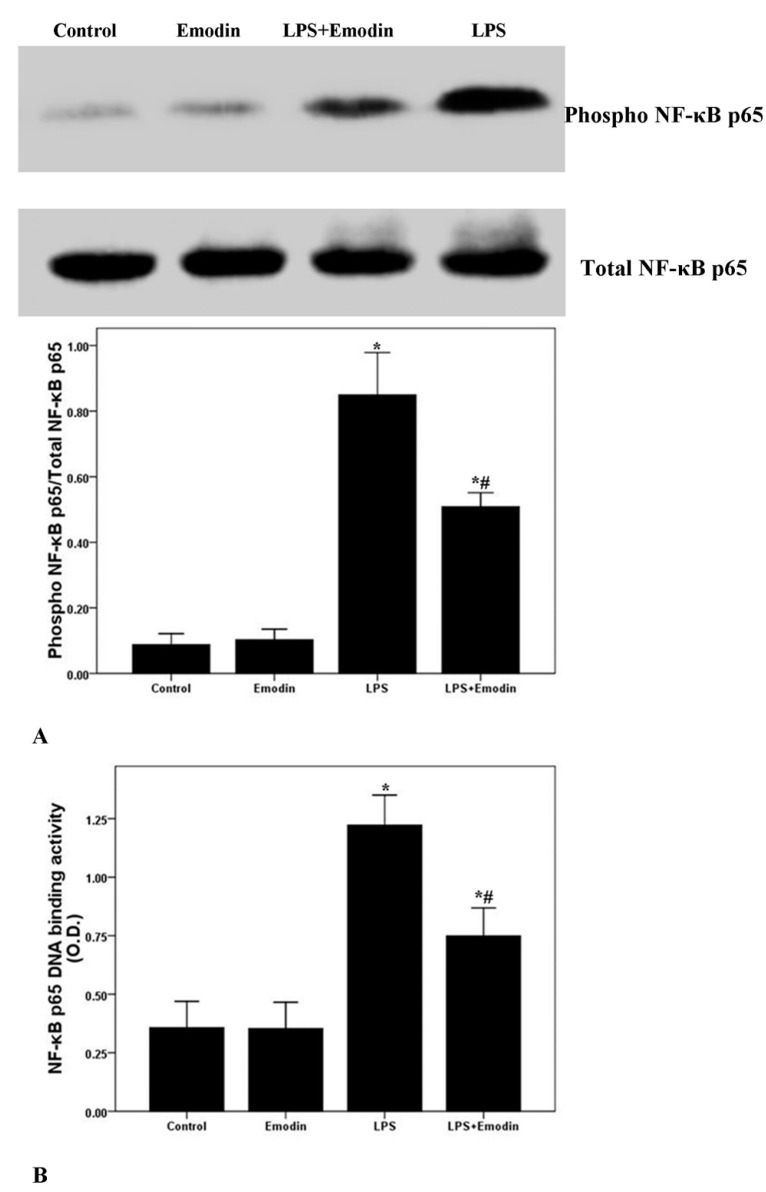
Emodin inhibits the phosphorylation and DNA binding activity of NF-κB p65 in LPS-induced ALI. After 72 h interventions, mice were exsanguinated and their lungs were removed. (**A**) Western blotting was used to analyze the phosphorylation of NF-κB p65 in lung; (**B**) DNA binding activity of NF-κB p65 was measured by a TranAM™ p65 transcription factor ELISA kit. Each bar represents the mean ± SD of 10 mice. * *p* < 0.05 compared with Control. # *p* < 0.05 compared with LPS.

NF-κB signaling pathway is essential for the regulation of LPS-induced inflammation and injury [15,29]. After 72 h of LPS injection, the phosphorylation and DNA binding activity of NF-κB p65 were markedly enhanced (Figure 5). However, LPS-induced phosphorylation and DNA binding activity of NF-κB p65 were notably inhibited by emodin treatment. Additionally, no difference in the phosphorylation and DNA binding activity of NF-κB p65 between the Control group and the Emodin group was observed.

## 3. Discussion

In the current study, our data revealed that LPS-induced pulmonary inflammation and pulmonary edema, and that the expression of E-selectin and MCP-1 were significantly inhibited by emodin, the active component of the herbal medicine *Radix rhizoma* Rhei, in mice. Furthermore, our results also suggest that this anti-inflammatory property of emodin possibly results from the inactivation of an NF-κB signaling pathway in LPS-induced ALI in mice.

ALI/ARDS results in severe lung disease or the pulmonary presentation of multiple organ dysfunction syndrome (MODS), with a high mortality rate from 30% to 50% in critically ill patients [1,2,3,14]. At present, ALI and its severe form ARDS, were defined as multiple intra-pulmonary or extra-pulmonary extreme conditions, such as severe pneumonia, lung abscess, severe sepsis, severe acute pancreatitis (SAP) and bone fractures, and induced uncontrolled and self-amplified pulmonary inflammation [1,2,3,14,15,16]. The studies confirmed that over-activated inflammatory cells (mainly containing neutrophils and macrophages) and their over expressed and/or released a variety of inflammatory mediators could directly and indirectly lead to alveolar epithelium and microvascular endothelium injuries in lung, which were the primary source of pathogenesis of ALI/ARDS [6,11,15,20]. Inflammation plays a hub role in ALI/ARDS [6,11,15,20]. Despite substantial progress being made in understanding of this disease, the available treatments are still limited in clinical practice and are mainly supportive treatment, such as mechanical ventilation and nutritional support, and etiological treatment. It is thus valuable and meaningful to find a new drug or treatment for ALI/ARDS.

Emodin, the bio-active compound of *Radix rhizoma* Rhei in traditional Chinese medicine, shows multiple pharmacological values, including anti-inflammatory, anti-fibrosis, anti-tumor proliferation, anti-atherosclerotic, immunosuppressive and anti-oxidative effects [22,23,24,25,26,27,28]. Li *et al.* found that LPS-induced mastitis in mice was markedly inhibited by emodin administrations [26]. And Chen *et al.* demonstrated that emodin demonstrated effective anti-fibrosis activity in bleomycin-induced pulmonary fibrosis in mice [28]. Yang *et al.* confirmed that high glucose-induced TGF-β1 and fibronectin (FN), the hallmarks in diabetic nephropathy, were notably attenuated by emodin in mesangial cells [25]. And in our study, LPS was used to induce the model of ALI/ARDS in mice. After 72 h of LPS stimulation, typical and severe pathological alterations were found, including notable inflammatory cell infiltration, interstitial and intra-alveolar edema and patchy hemorrhage, inter-alveolar septal thickening, hyaline membrane formation and some collapsed alveoli (Figure 1A). We also observed that these typical pathological changes and enhanced lung injury scores were both remarkably improved by emodin treatment (Figure 1A,B). At the early stage of the inflammation, pathogen-associated molecular patterns (PAMPs), such as LPS in gram-negative bacteria, proteoglycan in gram-positive bacteria and CpG DNA in pathogens, were recognized and bound by pattern recognition receptors (PPRs), including toll like receptors (TLRs), scavenger receptors (SRs) and mannose receptors (MRs), on inflammatory cells, particularly neutrophils and macrophages [30,31,32]. Meanwhile, it was confirmed that neutrophils and macrophages were mainly immunologic executive cells in LPS-induced pulmonary inflammation [15]. After the activation of inflammation, a variety of inflammatory mediators, such as TNF-α, IL-6, IL-1β and MCP-1, were synthesized and released into the tissues and blood, and expression of other inflammatory molecules, including E-selectin, P-selectin, VCAM-1 and ICAM-1, were up-regulated on inflammatory cells and tissue cells, particular on the vascular endothelium [15,29]. In the current study, we found that LPS significantly increased total cells, neutrophils and macrophages in BALF in mice. And these increments of inflammatory cells counts were largely reduced by emodin. Furthermore, our data showed that the LPS-induced up-regulation of TNF-α, IL-6 and IL-1β in BALF were also remarkably inhibited by emodin administration. Myeloperoxidase (MPO) was mainly synthesized and expressed by neutrophils, which is essential for the killing of phagocytosed pathogens, such as bacteria. Meanwhile, MPO activity was considered as the marker of the activation and accumulation of neutrophils in inflammation [15]. Our data demonstrated that MPO activity was largely raised after 72 h of LPS injection. However, this increase in MPO activity was significantly inhibited by emodin. Pulmonary edema without abnormal cardiac filling pressure, another major pathological feature of ALI/ARDS, resulted in decline of lung compliance and the failure of oxygenation, which was the main reason of death in patients with ALI/ARDS, particularly in the early stage [16]. In our study, we found that the LPS-increased W/D was significantly reduced by emodin treatment (Figure 1C). These findings indicated that the LPS-induced pulmonary inflammation and pulmonary edema were remarkably inhibited by emodin administration in mice.

Multiple reports confirmed that NF-κB was considered as one of the most important regulator in inflammation in ALI/ARDS [15,19,20]. The injury and inflammation of the lung tissues in ALI/ARDS can be attenuated by reduction or blocking the activation of NF-κB, which was proven both *in vivo* and *in vitro* [15,17,18,19,20]. NF-κB has been considered as a promising pharmacological target of inflammatory diseases, including sepsis, ALI/ARDS, asthma and arthritis. Several studies revealed that emodin was an effective inhibitor of the NF-κB signaling pathway [15,17,18,19,20]. Monocyte chemoattractant protein-1 (MCP-1), a CC chemokine also named as chemokine (C–C motif) ligand 2 (CCL2), was mainly expressed by alveolar macrophages and pulmonary vascular endothelial cells in the lung [33]. MCP-1 was quickly up-regulated by many stimuli, such as oxidative stress, inflammation and shear forces, leading to the recruitment of more inflammatory cells, particularly neutrophils and macrophages, infiltrating into the damaged tissues [33]. Several studies confirmed that MCP-1 played a key role in the pathogenesis of ALI/ARDS, especially at the initial phase [34,35]. It has been proven that two kappa B binding sites are located in the transcriptional regulatory area of the *MCP-1* gene [36]. Also, E-selectin is a critical adhesion molecule in the selectin family and only expressed on epithelium; it is essential for the mediation of epithelial cell binding to leukocytes with ESL-1, CD44 or PSGL-1, in inflammatory conditions [37]. Studies also confirmed that NF-κB lies at the center of the regulation of E-selectin expression [37,38,39]. For this reason, mRNA and protein expression of MCP-1 and E-selectin were analyzed in our study. We found that the expression of MCP-1 and E-selectin were noticeably up-regulated after 72 h of LPS stimulation. And the LPS-enhanced expression of MCP-1 and E-selectin were markedly inhibited by emodin intervention in mice. To gain insight into the anti-inflammatory properties of emodin, the activation of NF-κB p65 was evaluated by us. As shown in Figure 5, LPS-enhanced the phosphorylation of NF-κB p65, and NF-κB p65 DNA-binding activity was substantially inhibited by emodin administration in mice. These data suggested that the anti-inflammatory property of emodin was very likely mediated by inactivation of the NF-κB signaling pathway in LPS-induced ALI in mice.

## 4. Materials and Methods

### 4.1. Animals

All procedures involving animals were approved by the Committee on the Ethics of Animal Experiments of West China Medical School of Sichuan University. The present study was performed according to the recommendations in the Guide for the Care and Use of Laboratory Animals. All surgery was performed using sodium pentobarbital anesthesia, and all efforts were made to minimize suffering. Specific pathogen-free male BALB/c mice (16–20 g) aged 6 to 8 weeks were maintained under specific pathogen-free conditions in the animal center facilities of the our university. The mice were kept in a temperature-controlled room (12-h dark and light cycles) and offered *ad libitum* access to food and water. Animals underwent an acclimatization period of at least 7 days before study.

### 4.2. Murine Model of LPS-Induced ALI

Forty male BALB/c mice were randomly and evenly divided into 4 groups: Control group, Emodin group, LPS group and LPS + Emodin group. According to our previous report, ALI was induced by lipopolysaccharide (*Escherichia coli*, serotype 0111:B4; Sigma–Aldrich, St. Louis, MO, USA) via intratracheal injection [15]. Briefly, mice were anesthetized with 30 mg/kg of pentobarbital sodium, followed by 10 μg of LPS in 50 μL sterile saline intratracheal injection with a 3-gauge needle. The mice in the Control group were administrated the sterile saline instead. Then, the mice were placed in a vertical position and rotated for 1 min to distribute the instillation in lung. Ten minutes after the LPS injection or saline injection, mice were orally administered with emodin (Sigma, St. Louis, MO, USA) at 100 mg/kg every 12 h (7 times in all).

### 4.3. Bronchoalveolar Lavage Fluid (BALF) and Cells Counting

Seventy-two hours later, mice were sacrificed after anesthesia by pentobarbitone (50 mg/kg i.p.). According to our previous report, BALF was collected by cannulating the upper part of the trachea, by lavage 3 times with 1.0 mL PBS (pH 7.2). The fluid recovery rate was about 90%. Lavaged samples were kept on ice. BALF was centrifuged at 700× *g* for 5 min at 4 °C. The sediment cells were resuspended in 50 μL PBS and stained with Diff-Quik (International Reagents Corp., Kobe, Japan) for cytospin preparations. Then, total cells, neutrophils, macrophages and lymphocytes were counted double-blindly with a hemocytometer [17,40].

### 4.4. TNF-α, IL-6 and IL-1β in BALF

The BALF supernatant was collected after centrifugation (for 4 min at 4000 rpm) and stored at −80 °C before cytokine assay. TNF-α, IL-6 and IL-1β in BALF were measured by ELISA (R&D Systems, Minneapolis, MN, USA).

### 4.5. Myeloperoxidase (MPO) Activity Assay

MPO activity was measured according to our previous report [15]. Results are expressed as units of MPO activity per gram of lung tissue.

### 4.6. Lung Wet/Dry Weight Ratio

The severity of pulmonary edema was assessed by the wet to dry ratio (W/D) according to [15]. The left lower lungs were weighed and then dehydrated at 60 °C for 72 h in an oven.

### 4.7. H&E Staining

The right lower lung of each mouse was fixed in 10% formalin, embedded in paraffin, cut into 5 μm sections, and stained with H&E to analyze the pathological alterations of the lung tissues. Lung injury score was measured according to [15]. In brief, a score of 0 represented no damage, l represented mild damage, 2 represented moderate damage, 3 represented severe damage, and 4 represented very severe histologic changes.

### 4.8. Quantitative PCR

The mRNA expression levels of E-selectin and MCP-1 were detected by qPCR. And β-actin was included as internal reference. Briefly, the right upper lung tissues were kept in −80 °C, total RNA was isolated from the lung tissues by Trizol reagent (Invitrogen, Carlsbad, CA, USA). PrimerScript^®^ RT reagent Kit with gDNA eraser (Takara Bio Inc., Otsu, Japan) was used to the reverse transcription. Then, PCR was performed with a iQ™ 5 Multicolor Real-Time PCR Detection System (Bio-Rad Laboratories, Inc., Hercules, CA, USA) and a SYBR Green PCR kit (Takara Bio Inc.) in a final volume of 20 μL, containing 1.6 μL cDNA template, forward and backward primers 0.8 μL each, 10 μL SYBR^®^ Premix Ex Taq™ II and 6.8 μL dH_2_O. The primers and Taqman probes were designed using Primer Premier (PREMIER Biosoft International, Palo Alto, CA, USA). The premier sequences were as follows: E-selectin (forward) 5'-CATGACGTATGATGAAGC-3' and (reverse) 5'-GATTGGAGTTAAGGTAGTTG-3'; MCP-1 (forward) 5'-TTAAAAACCTGGATCGGAACCAA-3' and (reverse) 5'-GCATTAGCTTCAGATTTACGGGT-3' and β-actin, (forward) 5'-GATTACTGCTCTGGCTCCTAGC-3' and (reverse) 5'-ACTCATCGTACTCCTGCTTGCT-3'. Changes in the expression of target genes were calculated using the 2^−ΔΔ*C*t^ method, ΔΔ*C*_t_ = (*C*_t,target_ − *C*_t,β-actin_)_sample_ − (*C*_t,target_ − *C*_t,β-actin_)_control_ [41].

### 4.9. Western Blotting

According to the previous study, western blotting was performed to analyze the protein expression [18]. In brief, the proteins obtained from the left upper lung tissues were homogenized in PBS with protease inhibitor cocktail. The homogenates were centrifuged for 15 min at 14,000 rpm in 4 °C. Supernatants of the tissues were collected, and protein concentration was measured with a bicinchoninic acid assay kit using BSA as standard (Pierce, Rockford, IL, USA). An equal amount of protein from each sample (150 μg) was resolved in 10% Tris-glycine SDS polyacrylamide gel. Protein bands were blotted to nitrocellulose membranes. After incubation for 1 h in blocking solution at room temperature, the membrane was incubated for 24 h with anti-E-selectin (Santa Cruz Biotechnology, Inc., Santa Cruz, CA, USA), anti-MCP-1 (Santa Cruz Biotechnology, Inc.), anti-phospho-NF-κB p65 (Santa Cruz Biotechnology, Inc.), anti-NF-κB p65 (Santa Cruz Biotechnology, Inc.), or anti-β-actin (Santa Cruz Biotechnology, Inc.) at 4 °C, respectively. The secondary antibody (horseradish peroxidase-conjugated donkey anti-rabbit immunoglobulin) was added and incubated at room temperature for 1 h. Peroxidase labeling was detected with the enhanced chemiluminescence Western blotting detection system (Amersham Pharmacia Biotech, Piscataway, NJ, USA) and analyzed by a densitometry system. The relative protein levels of E-selectin and MCP-1 were normalized to β-actin. The levels of phospho-NF-κB p65 in each sample were measured as ratios of intensities of phospho-NF-κB p65 to total NF-κB p65 bands.

### 4.10. NF-κB p65 DNA Binding Activity Assay

According to our previous study, TranAM™ NF-κB p65 Chemi Transcription Factor Assay Kit was used (Active Motif, Carlsbad, CA, USA) to detect NF-κB p65 DNA-binding activity, following the instructions of the manufacturer [15].

### 4.11. Statistical Analysis

Statistical analyses were performed with SPSS software, version 17.0 (SPSS, Inc., Chicago, IL, USA). All data were presented as mean ± SD. Then, one-way analysis of variance (ANOVA) followed by Student’s *t*-test was used. *p* < 0.05 was considered to be statistically significant.

## 5. Conclusions

Taken together, our results indicated that emodin potently inhibited LPS-induced pulmonary inflammation, pulmonary edema and the expression of MCP-1 and E-selectin, and these effects were very likely mediated by inactivation of the NF-κB signaling pathway in mice. These results suggest a therapeutic potential of emodin as an anti-inflammatory agent in ALI/ARDS.
